# Antimicrobial, Antibiofilm, and Anti-persister Activities of Penfluridol Against *Staphylococcus aureus*

**DOI:** 10.3389/fmicb.2021.727692

**Published:** 2021-08-18

**Authors:** Yaqian Liu, Pengfei She, Lanlan Xu, Lihua Chen, Yimin Li, Shasha Liu, Zehao Li, Zubair Hussain, Yong Wu

**Affiliations:** Department of Laboratory Medicine, The Third Xiangya Hospital, Central South University, Changsha, China

**Keywords:** drug repurposing, penfluridol, methicillin-resistant *Staphylococcus aureus*, biofilm, bacterial persister, membrane permeability

## Abstract

*Staphylococcus aureus* has increasingly attracted global attention as a major opportunistic human pathogen owing to the emergence of biofilms (BFs) and persisters that are known to increase its antibiotic resistance. However, there are still no effective antimicrobial agents in clinical settings. This study investigated the antimicrobial activity of penfluridol (PF), a long-acting antipsychotic drug, against *S. aureus* and its clinical isolates *via* drug repurposing. PF exhibited strong bactericidal activity against *S. aureus*, with a minimum inhibitory concentration (MIC) and minimum bactericidal concentration (MBC) of 4–8 and 16–32 μg/ml, respectively. PF could significantly inhibit biofilm formation and eradicate 24 h preformed biofilms of *S. aureus* in a dose-dependent manner. Furthermore, PF could effectively kill methicillin-resistant *S. aureus* (MRSA) persister cells and demonstrated considerable efficacy in a mouse model of subcutaneous abscess, skin wound infection, and acute peritonitis caused by MRSA. Notably, PF exerted almost no hemolysis activity on human erythrocytes, with limited cytotoxicity and low tendency to cause resistance. Additionally, PF induced bacterial membrane permeability and ATP release and further caused membrane disruption, which may be the underlying antibacterial mechanism of PF. In summary, our findings suggest that PF has the potential to serve as a novel antimicrobial agent against *S. aureus* biofilm- or persister-related infections.

## Introduction

*Staphylococcus aureus* is an important opportunistic human pathogen that often causes disease in community and hospital settings (van Dalen et al., 2020). *Staphylococcus aureus* is both a frequent commensal and a leading cause of nosocomial infections, including bacteremia, osteomyelitis, endocarditis, and skin and soft tissue infections (Turner et al., 2019). With the widespread emergence of virulent and multidrug-resistant strains of methicillin-resistant *S. aureus* (MRSA; Miller et al., 2020), there is an urgent and unmet clinical demand for a novel approach to cure these infections as the increasing antibiotic resistance is recognized as a major threat to global public health (Gandra et al., 2020).

The National Institutes of Health estimates that more than 80% of bacterial infections are accompanied by biofilm (BF) formation, with approximately 17 million new biofilm-related infections in the United States annually (Joseph et al., 2016). Infections caused by biofilm-related pathogens are difficult to eradicate as they can transition from acute stages to chronic stages, causing serious complications (Abraham, 2016). *Staphylococcus aureus* is one of the most common pathogens of biofilm-related clinical infections, and can adhere to the surface of medical devices or human tissues to form biofilms (Arciola et al., 2018). Compared with planktonic bacteria, biofilm bacteria are much less susceptible to antibiotic and can evade the host’s immune system, leading to a prolonged infection. The extracellular matrix of the biofilm prevents antimicrobial from reaching the bacteria embedded in a biofilm, greatly increasing drug resistance of bacteria in the biofilm. Therefore, it is urgent to develop new antimicrobial drugs to combat biofilm-associated infection, as it is proving difficult for traditional antibiotics to effectively eradicate the biofilms formed in the body (Koo et al., 2017).

In addition to the well-known strategies of antimicrobial resistance and biofilm formation, bacterial colonies have an additional survival strategy that can endure harsh environment or antibiotic exposure. A small fraction of temporarily antibiotics-tolerant phenotypic mutants, known as persister cells, are capable of surviving from high dose of antibiotics treatment (Defraine et al., 2018). The presence of persisters can lead to recalcitrance and relapse of persistent bacterial infections, which further contribute to antimicrobial treatment failure (Fisher et al., 2017). Moreover, frontline MRSA treatments, including daptomycin (DAP) and vancomycin (VAN), have been unable to eradicate persisters (Garrison et al., 2015), highlighting the urgency of novel antibacterial therapeutics to tackle chronic infections and improve the prognosis of patients.

The decrease in new antimicrobial candidates coupled with the increase in antibiotic resistance makes it necessary to develop alternative antibacterial drugs. Drug repurposing is a promising alternative strategy (Nosengo, 2016), the major theoretical basis for reusing approved drugs is that they are equipped with known modes of action, pharmacological analysis, and controllable side effects, thereby reducing the risks, time, and costs involved in developing new drugs. An example is the derivatives of thalidomide, originally designed to treat morning sickness, which have had remarkable clinical success in the therapeutic field well-beyond their original approved use (Mori et al., 2018; Schein, 2020). Evidently, drug repurposing has a broad prospect of clinical application, and more novel antimicrobials can be discovered through this strategy.

Penfluridol (PF), an oral long-acting antipsychotic drug approved by the Food and Drug Administration, is used to treat chronic schizophrenia, acute psychosis, and Tourette’s syndrome (Soares and Lima, 2006). The mechanism of PF action against psychotic disorders is thought to involve the blocking of dopamine receptors, especially to postsynaptic D2 receptor (Tuan and Lee, 2019). It has been demonstrated that PF exerts significant antibacterial and biofilm eradicating effects against *Enterococcus faecalis* (Zeng et al., 2021). However, its effects on persister cells, antibacterial mechanism and therapeutic efficacy *in vivo* against *S. aureus* have not been systematically studied. In this study, we conducted a detailed bioanalysis of PF as an antibacterial agent, including its mechanism of action and therapeutic efficiency in subcutaneous abscess infection, skin wound infection, and acute peritonitis models of MRSA infection.

## Materials and Methods

### Bacterial Isolates, Growth Conditions, and Reagents

*Staphylococcus aureus* Newman, RJ-2, and ATCC 43300 were kindly provided by Li Min (Shanghai Jiaotong University, Shanghai, China). The type strains including *S. aureus* ATCC 29213, *E. faecalis* ATCC 29212, *Escherichia coli* ATCC 25922, *Acinetobacter baumannii* ATCC 1195, and *Klebsiella pneumoniae* ATCC 700603 were kindly given by Juncai Luo (Tiandiren Biotech, Changsha, China). *Staphylococcus epidermidis* ATCC 12228 and RP62A were obtained from Qu Di (Shanghai Medical College of Fudan University, Shanghai, China). *Pseudomonas aeruginosa* PAO1 was supplied by Qiao Minqiang (College of Life Sciences of Nankai University, Tianjin, China). Other clinical isolates strains were obtained from the Third Xiangya Hospital of Central South University (Changsha, China; She et al., 2020). *Staphylococcus epidermidis* and *S. aureus* were cultured in soybean trypsin broth (TSB; Solarbio, Shanghai, China). *Enterococcus faecalis* strains were grown in brain-heart infusion (BHI) broth (Solarbio, Shanghai, China); other gram-negative strains were cultured in Luria-Bertani (LB) broth (Solarbio, Shanghai, China). All bacteria were grown at 37°C, with shaking at 180–200 rpm. PF and other antibiotics were purchased from MedChem Express (New Jersey, United States).

### Antimicrobial Susceptibility Tests

The minimum inhibitory concentration (MIC) values of PF and other traditional antibiotics were determined by a broth microdilution method according to the recommendations of the Clinical & Laboratory Standards Institute (Harbut et al., 2015). Briefly, drugs were 2-fold diluted in Mueller Hinton (MH) broth (Solarbio, Shanghai, China) and mixed with an equal volume of approximately 1.5 × 10^6^ CFU/ml bacterial suspension into a microwell plate (Corning costar, United States) at 37°C for 18 h. MIC is defined as the lowest drug concentration with no visible bacterial growth. For the determination of the minimum bactericidal concentration (MBC; Li et al., 2020), bacterial suspension (10 μl) was plated on blood agar plate and incubated at 37°C for 24 h. MBC is considered as the minimum concentration without visible bacterial colonies growth on the plate after incubating at 37°C for 24 h.

### Killing Kinetics Assay

*Staphylococcus aureus* was inoculated into TSB medium and cultured overnight at 37°C and 180 rpm. The bacterial cultures were diluted with TSB medium containing PF at concentrations of 0.5–4× MIC, to the final bacterial concentration of 1 × 10^6^ CFU/ml; the bacterial culture was treated with 0.1% DMSO as a control. The suspensions were incubated at 37°C, 180 rpm, and samples, respectively were washed once with PBS, serially diluted, and plated on blood agar at 0, 2, 4, 6, 8, and 12 h. After incubating at 37°C for 24 h, the number of viable colonies was determined by plate counts (Thakare et al., 2017).

### Drug Combination Assay

Bacterial suspension at mid-log phase was diluted and distributed into 96-well microplates, and a two-dimensional chessboard was established by adding serial dilutions of PF and other antibiotics horizontally and vertically, with a final volume of 100 μl per well. Results were determined at OD_630_ using a microplate spectrophotometer (Bio-Rad, United States) after incubation at 37°C for 16–18 h. The fractional inhibitory concentration index (FICI) was calculated according to the following formula: FICI = MIC_A_ (combination)/MIC_A_ (alone) + MIC_B_ (combination)/MIC_B_ (alone). The FICI was judged as follows: FICI ≤ 0.5 indicates synergism; 0.5 < FICI ≤ 1 indicates additive; 1 < FICI ≤ 4 indicates irrelevant; and >4 indicates antagonism (MacNair et al., 2018).

### Biofilm Inhibition and Eradication Assay

*Staphylococcus aureus* cultured overnight was diluted 1:100 with fresh TSB culture medium, with or without antimicrobial agents, and 100 μl was added to a final drug concentration of 1–128 μg/ml in a microplate. After incubating at 37°C for 24 h, planktonic cells were gently washed twice with saline. The biofilm biomass was quantified with crystal violet staining. Briefly, 100 μl of 0.25% crystal violet was added to each well. After incubation at room temperature for 15 min, each well was washed using saline. Then, adding 100 μl 95% ethanol to dissolve stained dye for 20 min. The biofilm biomass was determined by measuring the absorbance at 570 nm with a microplate spectrophotometer. For biofilm eradication assay, the 24-h biofilms were constructed as mentioned above, treated with 100 μl of the various antimicrobial agents at 37°C for another 24 h, and then washed with saline; the biofilm biomass was determined by the CV staining method (She et al., 2019a).

### Biofilm-Forming Capacity

The 24 h biofilm was constructed in 96-well microplates as previously described; after incubation at 37°C for 24 h, biofilm biomass was measured by the CV staining method as described above, and the biofilm-forming ability was determined based on absorbance measured at 570 nm (A570_nm_). The biofilm-forming capacity was categorized according to the standard of Hassan et al. (2011), as follows: absorbance cut-off value (Ac) ≤ A570_nm_ ≤ 2 × Ac indicates non/weak biofilm production; 2 × Ac < A570_nm_ ≤ 4 × Ac indicates moderate biofilm production; and A570_nm_ > 4 × Ac indicates strong biofilm production, where Ac = average A570_nm_ of negative control + 3 × SD of negative control.

### Determination of Viable Cells in Biofilm

The 24-h mature biofilm was established as previously described. Thereafter, 100 μl saline was added to destroy the biofilm and mixed by strong pipetting to assure separation from the well. Bacterial mixtures were subsequently transferred to a fresh microplate, continuously diluted 10-fold in saline, and viable bacteria were counted by a plate counting method (She et al., 2019b).

### Confocal Laser Scanning Microscope Observation Analysis

A confocal laser scanning microscope observation (CLSM) was used to visually evaluate the effect of PF in eradicating biofilm. The overnight culture of *S. aureus* was 1:100 diluted with fresh TSB medium, and 2 ml of *S. aureus* cultures were subsequently added to the six-well microwell plate (Corning/Costar, United States), the sterile glass sheet was placed into the well to form a 24-h biofilm. Thereafter, incubation at 37°C for 24 h, the bacterial suspension was treated with 16 and 32 μg/ml PF, with no drug treatment or 64 μg/ml VAN as control. After incubation for 24 h, the mixture of SYTO9 and PI (Thermo Fisher Scientific, Shanghai, China) was used to dye the biofilm. The glass slides were visualized by a CLSM (ZEISS LSM800, Jena, Germany), and the biofilm biomass was quantified with ImageJ software (Liu et al., 2015).

### Persister Killing Assay

Persisters of *S. aureus* strains were prepared by culturing overnight to stationary phase at 37°C with shaking at 200 rpm (Kim et al., 2018). Overnight culture was washed three times with PBS and adjusted to OD_630_ = 0.2; then 2–8× MIC PF (8× MIC VAN as control) was added to 2 ml of the persister cell suspension and incubated at 37°C, 200 rpm. Samples were washed and harvested, respectively, at 0, 2, 4, and 6 h, and the total number of colonies was determined by plate count.

### Persister Membrane Permeability Assay

Persisters were obtained following a previous protocol (Kim et al., 2019); persister cells were washed three times and adjusted to OD630 = 0.4 with PBS. SYTOX Green (Thermo Fisher Scientific, United States) was added to 5 ml of this bacterial suspension to a final concentration of 5 μM and incubated in the dark for 30 min. The bacterial/SYTOX Green suspension (50 μl) was mixed with 50 μl of desired concentration of PF in black, clear-bottom microplates (Corning, United States). Fluorescence was measured using a microplate reader (PerkinElmer EnVision, United States) with excitation and emission wavelengths of 485 and 525 nm, respectively.

### Biofilm Persisters Killing Assay

To obtain persister cells from the biofilm, a mid-logarithmic phase culture of MRSA was diluted to 1 × 10^8^ CFU/ml in BHI broth. Diluted bacterial suspension (100 μl) was added to the 96-well plate, and the plate was sealed and incubated in a humid atmosphere at 37°C for 24 h. The plate was washed twice to remove planktonic bacteria, and biofilm was exposed to 100 μl BHI medium containing 100× MIC rifampin (RFP). After incubation at 37°C for 24 h, planktonic bacteria were removed, and any residual bacteria attached to the well were transferred into 100 μl saline by 5 min sonication. Bacteria were then treated with 200 μl of saline containing 2, 4, and 8× MIC PF. Bacteria were exposed to saline without antibiotics or with 64 μg/ml VAN, which served as a control. The number of live bacteria in biofilm was determined at 0, 2, 4, 6, and 24 h by plate count (de Breij et al., 2018).

### Drug Resistance Screening

The drug resistance of *S. aureus* to PF was evaluated by single-step drug resistance and sequential passaging resistance. For single-step frequency of resistance, overnight cultures of *S. aureus* ATCC 29213 and ATCC 43300 were adjusted to OD_630_ = 0.5 with TSB medium. Bacterial suspensions (100 μl) were subsequently spread on pre-prepared MH agar containing 2 and 4× MIC of RFP, ciprofloxacin (CIP), and PF; after 48 h of incubation, the drug spontaneous resistance frequency was defined as the number of resistant colonies divided by the initial viable cell count (Qu et al., 2020). For sequential passaging resistance, the MICs of PF and CIP against *S. aureus* were determined on the first day. After incubating for 16 h, bacterial suspension (5 μl) was taken from the well, with an antibiotic concentration of 0.5× MIC, and then diluted 1,000-fold with MH broth. Bacterial suspensions (50 μl) and 50 μl of diluted drugs were added to a 96-well plate, and the MIC was determined the next day. The scheme was successively performed for 25 days (Friedman et al., 2006).

### Membrane Permeability Assay

*Staphylococcus aureus* ATCC 29213 and ATCC 43300 were grown to mid-log growth phase, washed, and suspended in 5 mM HEPES (pH 7.2), and OD_630_ was adjusted to 0.05. SYTOX Green fluorescence dye was added to bacterial suspension to a final concentration of 2 μM, and then treated with various concentrations of PF. The intensity of fluorescence was measured for 20 min with the excitation and emission wavelengths of 485 and 525 nm, respectively (Zhou et al., 2020).

### Membrane Depolarization Assay

*Staphylococcus aureus* in the mid-log growth phase was washed and suspended in 5 mM HEPES, adjusted at OD_630_ to 0.05, incubated with 100 mM KCl, 5 mM glucose, and 2 μM Disc 3(5) (AAT Bioquest, United States) at room temperature in the dark atmosphere for 1 h, and serially diluted with PF to monitor the fluorescence intensity every 30 s for a total of 5 min. The excitation and emission wavelengths were at 622 and 670 nm, respectively. Melittin (10 μg/ml) and 0.1% DMSO were served as a positive and negative controls, respectively (Zhou et al., 2020).

### Total Reactive Oxygen Species Measurement

ROS levels in *S. aureus* ATCC 433300 treated by PF were detected with 2′,7′-dichlorofluorescein diacetate (DCFH-DA), according to the manufacturer’s instructions (Beyotime, Shanghai). Briefly, *S. aureus* was grown overnight at 37°C and 200 rpm, and then washed and suspended in 1× PBS with OD_630_ of 0.5. The bacterial suspension was incubated with DCFH-DA at a final concentration of 10 μmol/L for 30 min. After washing twice with PBS, 190 μl of bacterial cells labeled with probe and 10 μl of PF were then added to a microwell plate and incubated for another 30 min. The fluorescence intensity was immediately detected by a microplate reader at excitation wavelength of 488 nm and emission wavelength of 525 nm (Song et al., 2020).

### Measurement of ATP Release

Intracellular and extracellular ATP levels of *S. aureus* were measured by an Enhanced ATP Assay Kit (Beyotime, Shanghai, China). *S. aureus* ATCC 43300, cultured overnight, was washed and suspended with PBS to gain an OD_630_ of 0.5. After treatment with different concentrations of PF (0–16 μg/ml) for 1 h, the bacterial suspension was centrifuged at 12,000 × *g*, 4°C for 5 min; thereafter, the supernatant was harvested to measure the level of extracellular ATP. In addition, the bacterial sediments were cleaved by lysozyme and harvested the supernatant to determine the level of intracellular ATP. The supernatant was dispensed into the microplate and incubated for 5 min before recording luminescence intensity using the microplate reader (Liu et al., 2020).

### NPN Uptake Assay

N-phenylnaphthylamine (NPN) uptake assay was conducted to investigate membrane permeability. *S. aureus* ATCC 433300 was diluted to 2 × 10^6^ CFU/ml, and then NPN was added to the final concentration of 20 μM, followed by incubation with different concentrations of PF for 15 min. Triton X-100 (0.1%) was used as a positive control. The fluorescence intensity was recorded (excitation wavelength 340 nm; emission wavelength 405 nm) using the microplate reader (PerkinElmer EnVision, United States) to evaluate the uptake of NPN (Elliott et al., 2020).

### Bacterial Viability Assay

Dead bacteria induced by PF were assessed with Live/Dead BacLight bacterial viability kit (Thermo Fisher Scientific, Shanghai, China). Overnight bacterial culture of *S. aureus* ATCC 43300 was washed twice, suspended in 1× PBS, and adjusted to OD_630_ of 0.1. Bacterial suspension was treated with serially diluted PF (0, 4, 8, and 16 μg/ml) and incubated for 1 h. The bacterial cells were washed, collected, and suspended in 1× PBS. SYTO9 (3.34 mmol/L, 0.75 μl) and PI (20 mmol/L, 0.75 μl) mixture were added to each sample and incubated for 15 min. Thereafter, fluorescence images were obtained using a CLSM, and the percentage of red and green fluorescent particles was determined by ImageJ software (Song et al., 2020).

### Scanning and Transmission Electron Microscopy

*Staphylococcus aureus* was cultured to mid-log growth phase at 37°C, 180 rpm, suspended in PBS, and treated with PF of 8× MIC for 1 h; bacteria without drug treatment served as controls. Bacteria were harvested by centrifugation at 4,000 × *g* for 5 min and observed by scanning electron microscopy (SEM) and transmission electron microscopy (TEM; HITACHI, Tokyo, Japan; She et al., 2020).

### RNA Sequencing

Gene expression levels in *S. aureus* ATCC 43300 were detected by treatment with PF or 0.1% DMSO. Samples were harvested 1 h post-treatment and were independently prepared for RNA sequencing. E.Z.N.A Total RNA Kit II was used to extract the total RNA, and subsequent transcriptomic analysis was performed by the Shanghai Applied Protein Technology (Shanghai, China).

### Hemolysis Assays and Cytotoxicity

Human red blood cells were purchased from the Hemo Pharmaceutical & Biological Co (Shanghai, China), and centrifuged at 500 × g, 4°C for 5 min. The supernatant was discarded and washed twice with saline. About 100 μl red blood cell (RBC) suspension and 100 μl different concentrations of PF were added to the microplate to a final RBC concentration of 4%. RBC suspensions were treated, with 0.2% DMSO and 0.1% Triton X-100, used as negative and positive controls, respectively. After 1 h of incubation, the absorbance of the supernatant was measured at 570 nm (A570), hemolysis rate was calculated as follows (Tan et al., 2019):

$$\mathrm{Hemolysis}\;\left(\%\right)=\frac{A_{\mathrm{sample}}-A_{0.2\%\mathrm{DMSO}}}{A_{\mathrm{TritonX}-100}-A_{0.2\%\mathrm{DMSO}}}\times 100\%$$

The CCK-8 kit (Tongren, Japan) was used to evaluate the cytotoxicity of PF on human bronchial epithelial cell line HBE, human lung adenocarcinoma cell line A549, and human brain microvascular endothelial cell (HBMEC). HBE, A549, and HBMEC cells were cultured in Dulbecco’s modified Eagle’s medium with 10% FBS (Gibco). Then, 100 μl of log-phase cells was added to a 96-well plate with a final concentration of 5 × 10^3^ cells/well and then incubated at 37°C with 5% CO_2_ for 24 h to make cells adhere. The cells were then exposed to different concentrations of PF or 0.1% DMSO included as control. After incubation under normal tissue culture conditions for 8 h, the cell viability was detected with CCK-8 kit following the manufacturer’s instructions.

### Pharmacokinetic Analysis

A single dose of 10 mg/kg PF was administered to ICR female mice by subcutaneous, intraperitoneal, or tail intravenous injection (*n* = 4, mice per group). Plasma samples were collected from the three groups at different time points (5, 15, and 30 min, and 1, 2, 4, 8, 24, 48, and 72 h). The concentration of PF in mice plasma was detected by LC–MS, and pharmacokinetic parameters were calculated using WinNonlin 7.0.

### Animals

#### Murine Subcutaneous Abscess Model

Specific pathogen-free ICR female mice (Hunan, China), aged 8 weeks and weighing 24–26 g were used. Mice were anesthetized with 50 mg/kg sodium pentobarbital *via* intraperitoneal injection, and the dorsal hair was removed with an electric razor. Prior to injection, *S. aureus* ATCC 433300 cells were washed and resuspended in saline. A 50 μl bacterial suspension containing 1 × 10^7^ CFU cells was subcutaneously injected into the dorsum. One hour after infection, 100 μl PF (5, 10, and 15 mg/kg) was directly injected subcutaneously into the infected site. The vehicle (0.5% DMSO) was applied as a negative control and 10 mg/kg VAN as a positive control. After treatment for 2 days, mice were euthanized through cervical dislocation and the size of abscess was measured; the skin was excised and homogenized with saline. The number of viable cells was counted (Pletzer et al., 2018).

#### Skin Wound Infection Model

Non-specific pathogen BALB/c female mice (Hunan, China), aged 6 weeks and weighing 16–18 g were used, and a bacterial suspension of 10^8^ CFU/ml of *S. aureus* ATCC 433300 was prepared. Mice were anesthetized with sodium pentobarbital by intraperitoneal injection. Mouse backs were shaved off with electric razors and anesthetized, and then sterile needles were used to create a wound with an area of approximately 2 cm^2^. *S. aureus* suspension (50 μl) was then loaded onto the wounded skin. Approximately 2 h after infection, Glaxal Base Moisturizing Cream containing 0.25, 0.5, and 1.0% PF was applied to the skin. Meanwhile, the vehicle (1.0% DMSO) was applied to the skin as a control and 1.0% VAN prepared ointment was used in the positive control group. After treatment for 24 h, mice were euthanized, the skin excised, and homogenized with saline. The number of viable bacteria was counted by plate counting method. Infected skin wound tissues were preserved in 4% formalin (Sangon), sectioned, and stained with hematoxylin–eosin (HE) for histopathological analysis. The skin of MRSA-infected mice was homogenized and centrifuged, and the supernatant was stored at −20°C until analysis. The level of interleukin-6 (IL-6) was quantitatively detected with an ELISA kit according to the manufacturer’s instructions (Thangamani et al., 2016).

#### Mouse Acute Peritonitis Infection Model

To determine the toxicity *in vivo*, female ICR mice (*n* = 5) were injected intraperitoneally with 20, 40, 60, and 100 mg/kg PF. Survival rates of treated mice were recorded during 7 days. The mouse acute peritonitis model was generated as described previously (Su et al., 2019), with minor modifications. Briefly, *S. aureus* USA300 was washed and resuspended in saline, and prepared to a concentration of 1 × 10^7^ CFU/ml containing 5% mucoprotein (Cool Chemistry, Beijing, China) for subsequent use. Mice were injected intraperitoneally with 500 μl of the prepared bacterial suspension. Thereafter, groups of mice (*n* = 6) were intraperitoneally administered, at 1 and 12 h after infection, with 20 mg/kg PF, 20 mg/kg VAN, or saline with 0.6% DMSO, the latter two acting as a positive control or vehicle group, respectively. After infection for 24 h, mice were euthanized, and their liver, kidney, and spleen were excised, homogenized, serially diluted, and then the bacterial load was determined.

### Statistical Analyses

All experiments were performed with three biological replicates. GraphPad Prism (version 8.0) was used for statistical analysis. Unless otherwise noted, value of *p* were calculated using Student’s *t*-test for two group comparisons or one-way ANOVA for comparisons among multiple groups. Value of *p* < 0.05 was considered to indicate a statistically significant difference. ^*^p < 0.05; ^**^p < 0.01; ^***^p < 0.001; and ^****^p < 0.0001.

## Results

### Antimicrobial Effects of PF Against *S. aureus*

Penfluridol exhibited strong antibacterial activities against type and clinical strains of *S. aureus*, *S. epidermidis*, and *E. faecalis*, with an MIC and MBC of 4–8 and 16–32 μg/ml, respectively. Similarly, we chose VAN and DAP as control to compare their antibacterial activity, because they are first-line antibiotics in the treatment of *S. aureus*-associated infection. The MICs of VAN and DAP against types and clinical strains of *S. aureus*, *E. faecalis*, and *S. epidermidis* were 1–4 and 0.5–8 μg/ml, respectively. The MBCs against *S. aureus*, *E. faecalis*, and *S. epidermidis* were 1–16 and 2–32 μg/ml for VAN and DAP, respectively. No antibacterial activities were observed for PF, VAN, and DAP against Gram-negative bacteria, including *K. pneumoniae* and *E. coli* with MICs >128 μg/ml (Table 1).

**Table 1:** Minimum inhibitory concentration (MIC) and minimum bactericidal concentration (MBC) determination of PF, VAN, and daptomycin (DAP) toward bacterial strains.

Strains	Penfluridol (μg/ml)		Vancomycin (μg/ml)		Daptomycin (μg/ml)	
	MIC	MBC	MIC	MBC	MIC	MBC
*Staphylococcus aureus*						
MSSAATCC29213	4	16	2	4	0.5	1
Newman	4	16	1	2	2	4
MRSA						
ATCC43300	8	32	2	4	0.5	2
MW2	4	16	2	2	1	4
USA300	4	16	1	1	1	4
RJ-2	4	32	1	4	2	4
SAJ1	8	32	4	8	4	4
*Staphylococcus epidermidis*						
ATCC12228	8	32	2	2	1	4
RP62A	8	32	4	4	4	8
Se1	4	32	1	1	2	2
*Enterococcus faecalis*						
ATCC29212	8	32	4	16	4	8
EF02	4	16	2	8	8	32
EF05	8	32	4	16	8	32
*Escherichia coli*						
ATCC25922	>128	>128	>128	>128	>128	>128
*Klebsiella pneumoniae*						
ATCC700603	>128	>128	>128	>128	>128	>128
*Acinetobacter baumanii*						
ATCC1195	>128	>128	>128	>128	>128	>128
*Pseudomonas aeruginosa*						
PAO1	>128	>128	>128	>128	>128	>128

MSSA, methicillin-sensitive *S. aureus*; and MRSA, Methicillin-resistant *S. aureus*.

**Figure 1:**
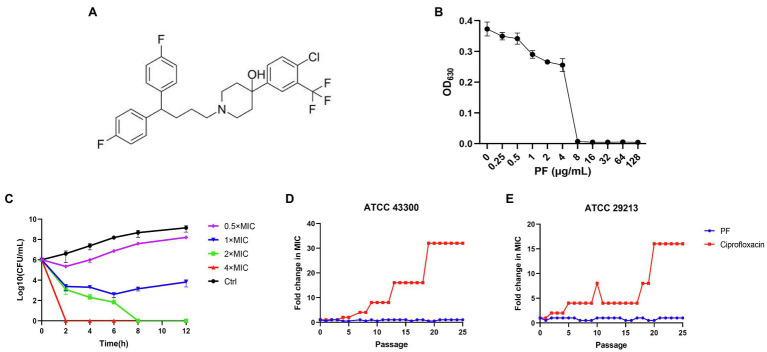
Penfluridol (PF) shows bactericidal activity against *Staphylococcus aureus* and without detectable resistance development. **(A)** The chemical structural formula of PF. **(B)** The growth of *S. aureus* ATCC 43300 after exposure to PF for 16–18 h. **(C)** Time killing curve of PF against *S. aureus* ATCC 43300, *S. aureus* was treated with 0.5–4× MIC of PF or 0.1% DMSO (control). Samples were harvested and counted at 0, 2, 4, 6, 8, and 12 h, the experiments were conducted triplicate and presented as mean ± SD. Sequential passaging resistance development of **(D)** *S. aureus* ATCC 43300 and **(E)** ATCC 29213 to PF and ciprofloxacin (CIP).

The chemical structural formula of PF was presented in the Figure 1A, the MIC of PF against *S. aureus* ATCC 43300 was 8 μg/ml (Figure 1B). To study whether the bactericidal effect of PF against MRSA ATCC 43300 was in a dose- and time-dependent manner, a time killing curve was generated (Figure 1C). Strong bactericidal effect was observed with PF concentrations at 1× MIC, and decreased bacterial cell counts from 6 to 2.6-log10 CFU/ml after treatment for 6 h. In addition, PF could rapidly kill all viable bacteria after treatment with 4 and 2× MIC for 2 and 8 h, respectively.

Checkerboard combination assay was conducted to investigate the effect of PF combined with several antibiotics against *S. aureus* ATCC 43300; results showed that, when combined with tetracycline (TC), the MIC of PF reduced from 8 to 2 μg/ml, while that of TC decreased from 2 to 0.5 μg/ml (Supplementary Figure 1A). Combined with 64 μg/ml polymyxin B nonapeptide (PMBN), the MIC of PF decreased by 4-fold, from 8 to 2 μg/ml (Supplementary Figure 1B). In addition, when PF was combined with fosfomycin (FOS), ceftriaxone (CRO), or levofloxacin (LEV), FICI was greater than 0.5, indicating an additive effect (Supplementary Figures 1C–E). The MIC of gentamicin (GN) did not decrease when combined with PF, and the FICI was calculated as >1, which was an irrelevant effect (Supplementary Figure 1F; Supplementary Table 1).

### Resistance Development Evaluation of PF to *S. aureus*

The results of sequential passaging resistance are demonstrated in Figures 1D,E. Serial passage of *S. aureus* ATCC 29213 and ATCC 43300 in existence of 0.5× MIC PF over a period of 25 days failed to produce drug resistance mutations as well, whereas the relative MIC values of CIP increased by 32 and 16-fold, respectively. With regard to single-step resistance evolution, the spontaneous resistance frequencies of PF were far below than that of CIP and RFP (Table 2). The spontaneous resistance frequencies of *S. aureus* ATCC 29213 and ATCC 43300 treated with 4× MIC PF were 4.16 × 10^−10^ (±7.21 × 10^−10^) and 8.77 × 10^−10^ (±1.52 × 10^−9^), respectively, while treatment with 4× MIC RFP yielded 6.67 × 10^−9^ (±1.91 × 10^−9^) and 2.10 × 10^−8^ (±5.25 × 10^−9^) frequencies, respectively. These results suggested that regardless of the short-term or long-term evolution of resistance, PF was better than RFP and CIP in reducing drug resistance mutations.

**Table 2:** Spontaneous resistance frequencies of PF for *S. aureus*.

Strains	Antimicrobial	Spontaneous resistance frequency (±SD)	
		2× MIC	4× MIC
ATCC 43300	PF	2.63 × 10^−9^ (±2.63 × 10^−9^)	8.77 × 10^−10^ (±1.52 × 10^−9^)
	Rifampin	1.06 × 10^−7^ (±9.35 × 10^−9^)	2.10 × 10^−8^ (±5.25 × 10^−9^)
	Ciprofloxacin	6.63 × 10^−7^ (±2.39 × 10^−8^)	1.66 × 10^−8^ (±4.01 × 10^−9^)
ATCC 29213	PF	1.66 × 10^−9^ (±2.88 × 10^−9^)	4.16 × 10^−10^ (±7.21 × 10^−10^)
	Rifampin	1.08 × 10^−8^ (±5.02 × 10^−9^)	6.67 × 10^−9^ (±1.91 × 10^−9^)
	Ciprofloxacin	3.13 × 10^−7^ (±1.40 × 10^−8^)	3.75 × 10^−9^ (±3.30 × 10^−9^)

### Biofilm Formation Inhibition and Eradication Effects of PF Against *S. aureus*

**Figure 2:**
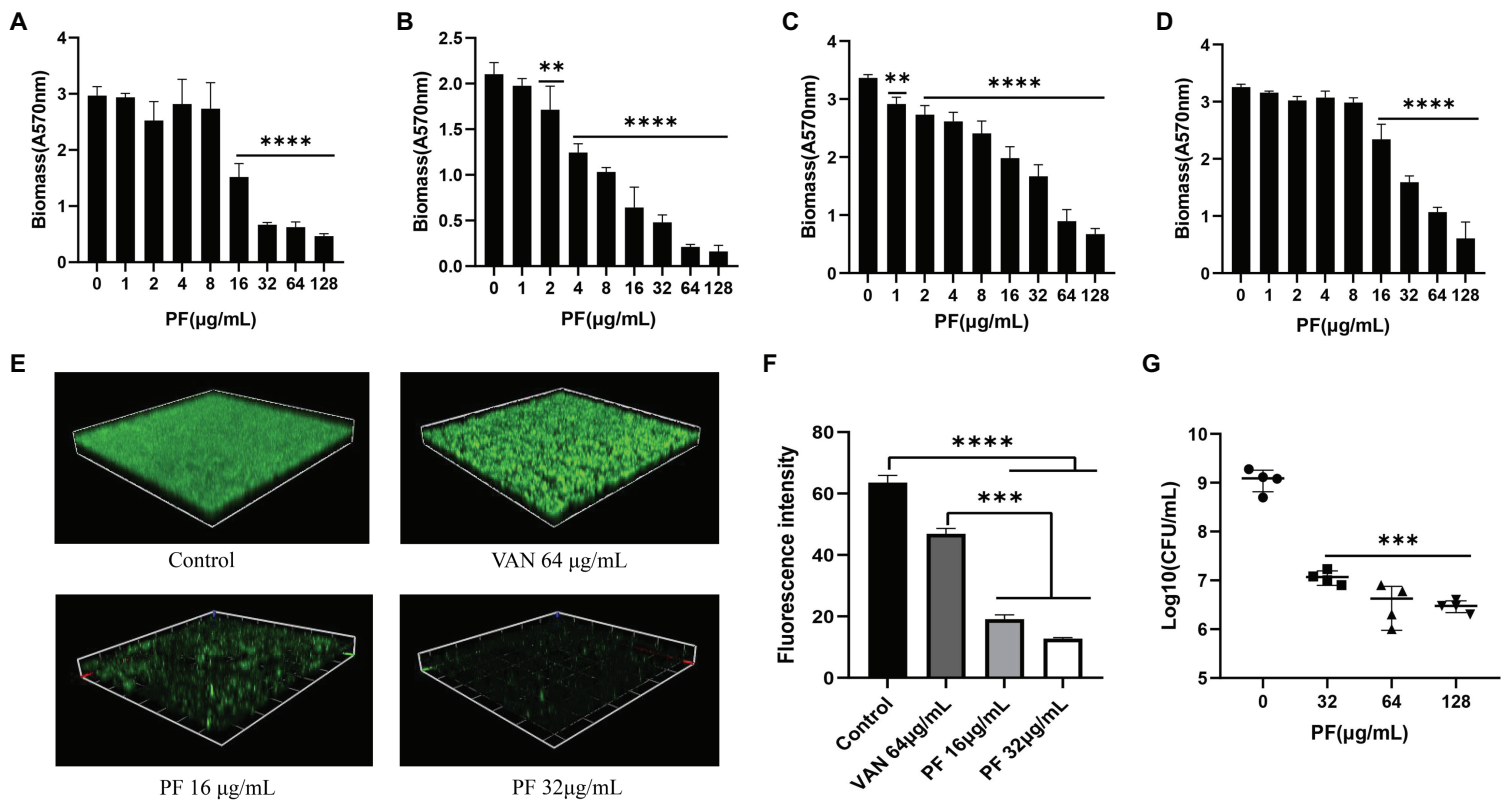
Biofilm eradication effect of PF against *S. aureus*. Biofilm-eradicating effects of PF against four strong biofilm producers **(A)** ATCC 43300 **(B)** USA 300 **(C)** SA 1901, and **(D)** LZB1, was measured by the crystal violet staining. **(E)** Confocal laser scanning microscope observation (CLSM) images (Scale bars, 40 μm) and **(F)** fluorescence intensity quantification analysis of biofilm-eradicating effect of PF against *S. aureus* ATCC 43300, by ImageJ software. **(G)** Live cells in biofilm of *S. aureus* ATCC 43300 were counted by colony counting method. Results were presented as mean ± SD. ^**^p < 0.01; ^***^p < 0.001; ^****^p < 0.0001.

The biofilm-forming ability of *S. aureus* (including MSSA and MRSA strains) is presented in Supplementary Table 2; four strains with strong biofilm formation capacity were picked for the biofilm eradication assay. PF could dose-dependently inhibit *S. aureus* biofilm formation when the concentration was ≥0.5 μg/ml. In addition, PF (*p* < 0.001) showed stronger inhibitory effects on biofilm formation than VAN at 0.5 μg/ml concentration (Supplementary Figures 2A,B). Similarly, PF demonstrated an effective biofilm-eradicating effect against four strains of *S. aureus* in a dose-dependent fashion (Figures 2A–D), in contrast to VAN, which could not eradicate biofilm (*p* > 0.05; Supplementary Figure 2C). The CLSM images also showed that 16 and 32 μg/ml PF exerted more significant biofilm eradication activities compared with 64 μg/ml VAN, with the green fluorescence signal intensity of viable bacteria markedly decreased and the density and thickness of biofilm significantly weakened (Figure 2E), as validated by the results of fluorescence intensity quantitative analysis (Figure 2F). The results of live bacteria count showed that, in contrast to the control, the number of live bacteria in the biofilm was significantly reduced in a dose-dependent fashion after treatment with PF (Figure 2G).

### PF Shows Bactericidal Activity Against MRSA Persister Cells

**Figure 3:**
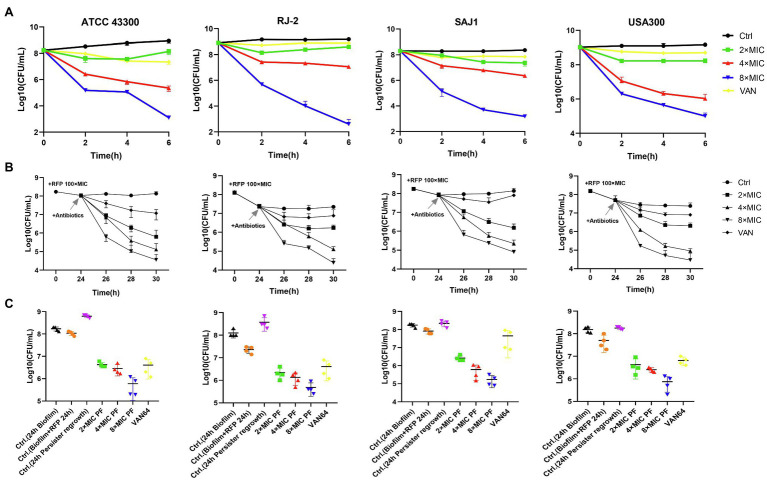
Penfluridol shows strong bactericidal activity against methicillin-resistant *S. aureus* (MRSA) persisters. **(A)** Viability of four MRSA persister cells exposed to 2–8× MIC PF for 6 h, untreated, or 8× MIC vancomycin (VAN) as control. Biofilm persisters were induced by rifampicin (RFP) at 100× MIC, then treated with 2–8× MIC PF and 64 μg/ml VAN for **(B)** 6 h, and **(C)** 24 h, respectively. Four strains of MRSA, from left to right, are ATCC 43300, RJ-2, SAJ1, and USA300, respectively. All the experiments were conducted triplicate; error bars represent means ± SD.

Four strains of MRSA with strong biofilm-forming capacity were used for the killing assay of persister cells. PF with 8× MIC could kill 4–6 log10 of persister cells, more effective than the 8-fold MIC of VAN (Figure 3A). For example, for the MRSA strain RJ2, the PF treatment of 4× MIC reduced the number of persister cells by 2.13 log10, 8× MIC PF decreased the cell number by 6.58 log10, whereas treatment with 8× MIC VAN did not exhibit bactericidal activity. Using a persister membrane permeability assay, performed with nucleic acid dye SYTOX Green, we observed that PF could induce dose-dependent membrane permeability in stationary-phase MRSA ATCC 43300 persister cells (Supplementary Figure 2D). It is suggested that the bactericidal activity of PF against persister cells may be due to its membrane-disrupting activity.

To evaluate PF activity against persisters derived from biofilm, we exposed biofilms of MRSA to a high dose of RFP (100× MIC) for 24 h. The bacteria wrapped in biofilms that survived after treatment with RFP, called persisters, were efficiently eradicated by PF. As shown in Figure 3B 2× MIC of PF treatment could kill nearly 10–100-fold number of persisters, 4× MIC of PF treatment decreased the number by 2–3 log10, and by 3–3.5 log10 when simultaneously treated with 8× MIC of PF, within 6 h; VAN (64 μg/ml) reduced the number of persister cells less than 10-fold compared to untreated persisters. Furthermore, the number of regenerated persister cells after 24 h of antibiotics treatment was slightly more than the initial bacterial count (Biofilm + RFP 24 h), and the germicidal efficacy of persisters treated with 8× MIC PF was still the most significant (Figure 3C). These results suggested that PF effectively killed MRSA planktonic and biofilm persister cells in a dose-dependent and time-dependent manner.

### Antibacterial Mechanism of PF Against *S. aureus*

**Figure 4:**
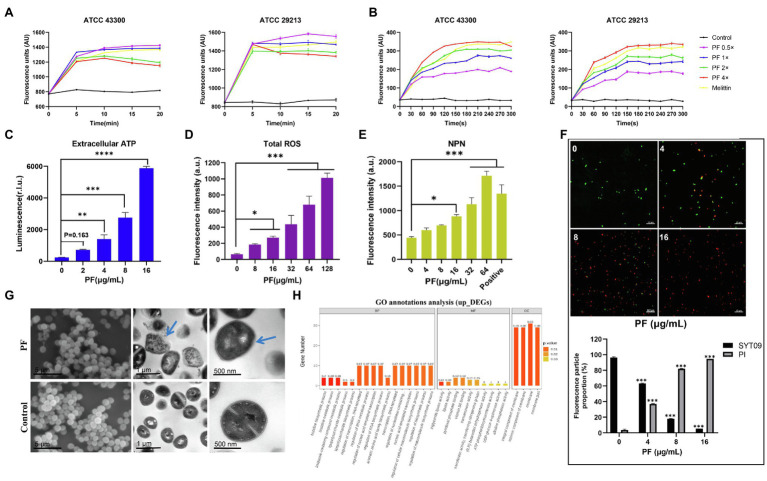
Mechanism of the antibacterial effect of PF against *S. aureus*. *Staphylococcus aureus* ATCC 29213 and ATCC 43300 were treated with various concentrations of PF, and the fluorescence intensity of **(A)** SYTOX Green and **(B)** Disc3(5) was measured by a microplate reader. **(C)** Increased levels of extracellular ATP in *S. aureus* ATCC 43300 after the treatment with PF. **(D)** Total ROS accumulation after treatment with PF. **(E)** Detection of membrane permeability with N-phenylnaphthylamine (NPN) probe. **(F)** Confocal images of *S. aureus* ATCC 43300 exposed to PF (0–16μg/ml) for 1h; live bacteria were stained green by SYTO9, whereas dead bacteria were stained red by PI. **(G)** Scanning electron and transmission electron microscopy (TEM) images of *S. aureus* ATCC 43300 treated with 64 μg/ml PF. Blue arrows represent areas with significant morphological changes. Scale bars, 5 μm (left), 1 μm (middle), and 100 nm (right). **(H)** GO annotation analysis of upregulated differentially expressed genes (DEGs) in *S. aureus* ATCC 43300 after exposure to PF for 1 h. ^*^p < 0.05; ^**^p < 0.01; ^***^p < 0.001; ^****^p < 0.0001.

To study the antimicrobial mechanism of PF against *S. aureus*, SYTOX Green was applied to detect the membrane integrity. Treatment of *S. aureus* ATCC 29213 and ATCC 43300 with different concentrations of PF resulted in a rapid increase in fluorescence intensity within 5 min (Figure 4A). When cells were treated with 0.5× MIC PF, membranes of *S. aureus* were efficiently disrupted, causing visible levels of dye uptake higher than those of the melittin-treated group (melittin is generally recognized as a membrane disruptor; Kaplan et al., 2011). The fluorescence intensity of PF with 1× MIC was comparable to that of melittin. However, the permeabilization decreased slightly at high concentrations of PF, which might be possibly attributed to the displacement of SYTOX Green from nucleic acid by PF, resulting in a reduction in its fluorescence emission intensity (Dias et al., 2017). Besides, we used the fluorescent dye Disc3(5), which is particularly sensitive to membrane potential changes, to observe the depolarization effects. We found that treatment of *S. aureus* with 4× MIC PF led to a rapid increase in fluorescence intensity (300–350 AU; Figure 4B), similar to that after melittin treatment. These results suggested that PF, which was as effective as melittin, penetrated the membrane of *S. aureus*, causing changes in membrane potential.

Furthermore, we observed an increase in extracellular ATP in *S. aureus* ATCC 43300 in a dose-dependent fashion after PF treatment (Figure 4C); intracellular ATP release is shown in Supplementary Figure 2E. In addition, PF induced the accumulation of ROS (Figure 4D). Accordingly, we next evaluated the impact of PF on the membrane permeability of *S. aureus*. Increased cell membrane permeability was observed based on the dose-dependent uptake of NPN (Figure 4E), and was further demonstrated by the increase in the number of bacteria from viable (green) to dead (red; Figure 4F). To visually observe bacterial membrane disruption by PF, SEM, and TEM were conducted. SEM showed that untreated *S. aureus* cell surface was smooth, without discernible ultrastructural changes, whereas bacterial cells treated with PF aggregated into clusters with filamentous adhesion and showed cell surface roughness with dimpled and deformed appearance. TEM showed that after treatment with PF, the boundary of bacterial membrane became blurry, the cell membrane was dissolved, and intracellular contents were leaked. In contrast, untreated cells showed smooth, clearly visible structures, with distinct and intact membranes (Figure 4G). Altogether, these data demonstrated that PF caused membrane disruption in *S. aureus*.

To have a better understanding of the molecular mechanism underlying the action of PF and the induced changes in gene expression at mRNA level, we further performed transcription analysis of *S. aureus* ATCC 43300 after exposure to PF for 1 h. Sequencing analysis exhibited significant transcriptional changes after treatment with PF. A total of 138 differentially expressed genes (DEGs) were observed in the PF-treated group, compared to controls (Supplementary Figure 2G). Of these, 80 were upregulated (including *vraX*, *ydfJ*, *hisZ*, *icaR*, etc.) and 58 were downregulated (*arcA*, *arcB*, *arcD*, and *fnbA*, etc.; Supplementary Figure 2F). GO annotation analysis identified alterations in the biological process (BP), molecular functions (MF), and cellular composition (CC), which showed that the upregulated DEGs were mainly correlated with membrane, intrinsic component of membrane, and integral component of membrane (Figure 4H). It is plausible that PF resulted in membrane disruption, which was compensated by the upregulation of membrane-related genes. Meanwhile, the downregulated DEGs were involved in catalytic activity, molecular function, and small molecule metabolic process (Supplementary Figure 2H). Moreover, KEGG enrichment analysis revealed that these DEGs were enriched in histidine, pyrimidine, tyrosine, cysteine, and methionine metabolisms (Supplementary Figure 2I).

### Toxicity and Pharmacokinetic Analysis

**Figure 5:**
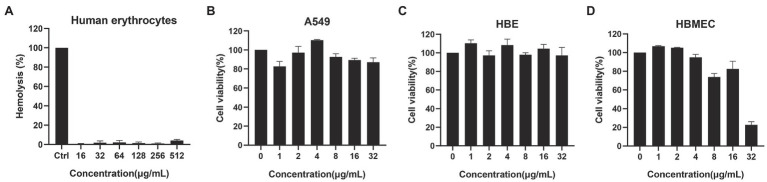
Hemolytic activity and cytotoxicity. **(A)** Human blood hemolytic activity of PF, 0.2% DMSO (negative control), and 0.1% Triton X-100 (positive control). The cytotoxicity of PF to **(B)** A549 **(C)** HBE, and **(D)** HBMEC cell lines was determined by CCK-8 assays.

**Figure 6:**
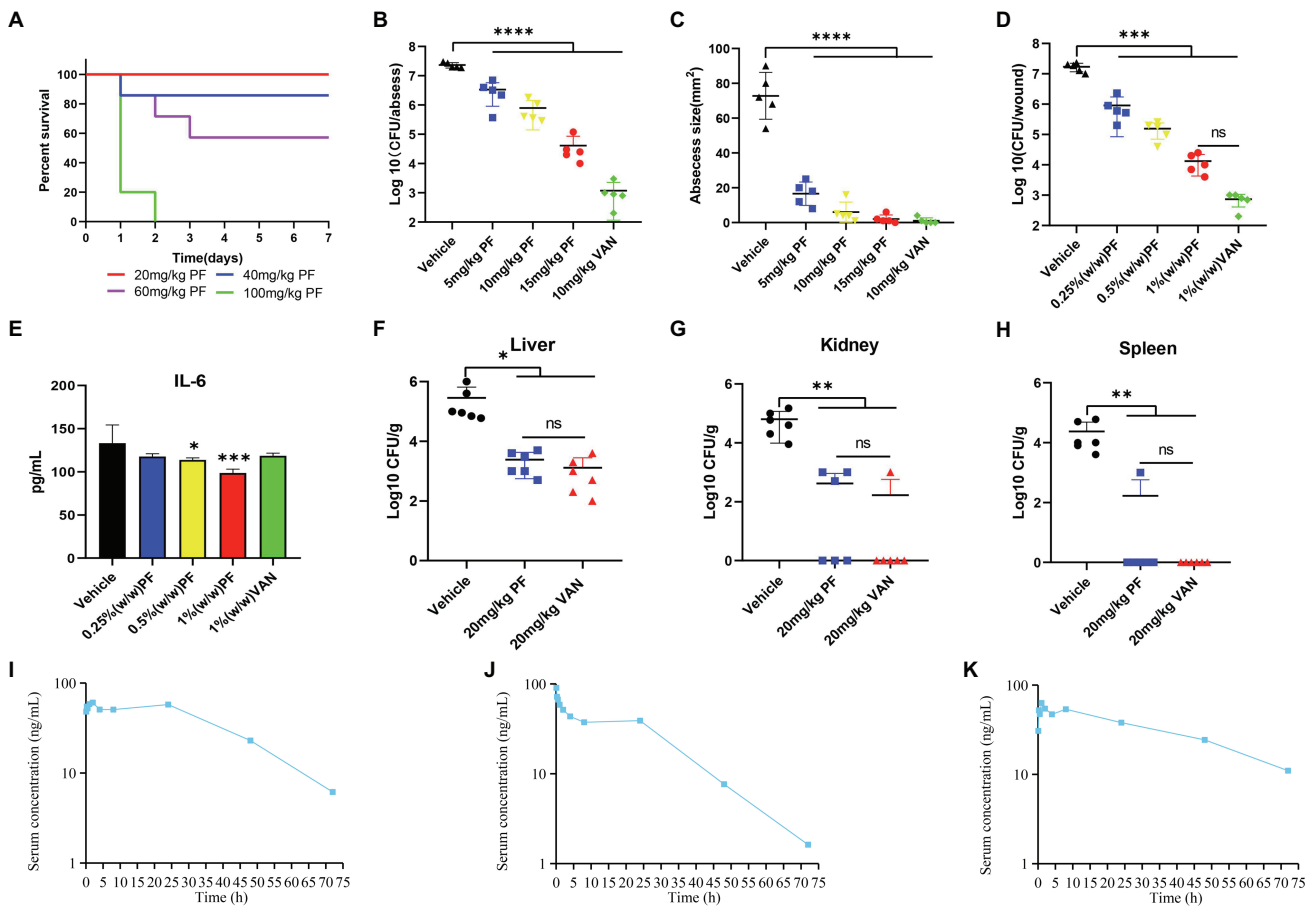
Penfluridol displays efficacy in murine local and systemic models of infection. **(A)** Toxicity test *in vivo*; mice (*n* = 5) was intraperitoneally injected with 20, 40, 60, and 100 mg/kg PF. PF showed significant effect on reducing the **(B)** bacterial load and **(C)** abscess area in a mouse subcutaneous abscess model. Antibacterial effect of an ointment containing PF on acute wound infection model **(D)** wound bacterial load, and **(E)** cytokine IL-6 levels. The number of viable colonies in the **(F)** liver **(G)** kidney, and **(H)** spleen was counted in a mouse acute peritonitis model. Quantification of PF plasma concentrations in mouse blood during 72 h after **(I)** intravenous **(J)** intraperitoneal, and **(K)** subcutaneous administration at a dose of 10 mg/kg (*n* = 4). ^*^p < 0.05; ^**^p < 0.01; ^***^p < 0.001; ^****^p < 0.0001.

Hemolysis assay showed that even when the concentration of PF was as high as 512 μg/ml, it could hardly cause hemolysis of human RBCs (Figure 5A). In addition, when the cytotoxicity of PF on HBE and A549 cell lines was assessed by a CCK-8 kit, almost no cytotoxicity of PF was detected at a dosage of 32 μg/ml, which was higher than the concentration at which the drug exerted antibacterial activity (4–8 μg/ml). Meanwhile, PF did not cause significant death of HBMEC cells at the value of MIC (Figures 5B–D). In addition, blood analysis after intravenous administration of PF at a single dose of 10 mg/kg showed that the half-life of PF was 15.74 h (Figure 6I). Similarly, the half-life of PF after intraperitoneal and subcutaneous administration at a dose of 10 mg/kg was 14.58 and 36.86 h, respectively (Figures 6J,K).

### PF Displays Efficacy in Murine Models of Infection

To test whether PF was effective in reducing topical and systemic infection of *S. aureus*, we used a skin abscess and wound infection model of local infections, as well as a peritonitis systemic infection model; all models were performed with an MRSA strain. The survival rate curve of mice after treatment with PF during 7 days is presented in Figure 6A. The mouse subcutaneous abscess model was constructed by subcutaneously injecting approximately 1 × 10^7^ CFU/mouse of MRSA ATCC 43300; the antibacterial efficiency *in vivo* was evaluated by subcutaneously administrating one dose of PF. Results showed that PF had significant antimicrobial effects, as confirmed by the reduced bacterial load of 0.84, 1.47, and 2.75 log10 CFU/abscess, when treated with 5, 10, and 15 mg/kg, respectively (Figure 6B). Moreover, treatment with PF (ranging from 5 to 15 mg/kg) could prominently inhibit abscess formation in a dose-dependent manner (Figure 6C).

To evaluate the potential of PF as a topical antimicrobial preparation, mice were infected with MRSA ATCC 43300 to form an open wound. After PF treatment, compared to the vehicle group, the number of bacteria from the infected skin lesions was significantly reduced. Mice treated with 1% VAN ointment showed the most significant reduction in bacterial load (4.26 log10 CFU/wound), followed by mice receiving 1% PF (3.11 log10 CFU/wound), 0.5% PF (2.04 log10 CFU/wound), and 0.25% PF (1.27 log10 CFU/wound). However, there was no statistical difference (*p* > 0.5) in the bacterial load of the mice skin treated with 1% PF and 1% VAN ointment (Figure 6D). Furthermore, we aimed to assess the effect of PF in controlling the inflammatory process associated with skin infection by MRSA by measuring pro-inflammatory cytokines (IL-6). As shown in Figure 6E, the 0.5% (*p* < 0.5) and 1% (*p* < 0.001) PF ointments reduced the cytokine IL-6 levels in the wounds of infected mice. The skin histopathology further showed that PF treatment could reduce the inflammation phenomena such as granulocyte infiltration (Supplementary Figure 3). This dual antimicrobial and anti-inflammatory activity of PF suggests its potential as a new local therapeutic medicine for MRSA-infected wounds.

In the systemic mouse peritonitis infection model, application of PF strongly decreased the bacterial load in the liver, kidney, and spleen. At 1 and 12 h post-infection, compared to the vehicle group, mice treated with 20 mg/kg PF and VAN showed significantly decreased bacterial load in the liver by 2.07 and 2.34 log10, respectively (Figure 6F), while treatment with 20 mg/kg PF and VAN showed 2.18 and 2.57 log10 reduction in the number of viable colonies in the kidney (Figure 6G). In addition, except for one mouse whose bacterial load decreased by 1.37 log10, the number of viable bacteria in the spleen of all other mice treated with PF decreased to 0 (Figure 6H). Most importantly, there was no significant difference (*p* > 0.5) in the bacterial load in the liver, kidney, and spleen between PF-treated and VAN-treated mice, both of which showed excellent antibacterial efficacy *in vivo*.

## Discussion

*Staphylococcus aureus* is an important human pathogen, which can colonize different tissue types and induce serious diseases in immunocompromised and other healthy individuals (DelMain et al., 2020). Treatment of *S. aureus*-related infections is challenging due to the rapidly evolving resistance mechanisms. In this study, we found that the antipsychotics drug PF exhibited strong antimicrobial effects against *S. aureus*. This study also showed that PF dramatically killed persister cells, whether non-biofilm or biofilm persisters, prevented biofilm formation and eradicated well-established biofilm. These results suggested that PF provides a feasible pathway toward developing treatments to cure chronic infections caused by biofilm and persisters. To our knowledge, this is the first study to report on the effect of PF on *S. aureus* biofilm and persisters, as well as the mechanisms underlying the antibacterial activities of PF.

Bacterial cell membrane is an attractive target for developing novel antibacterial drugs; many studies have proved that repurposed medicines, such as the anticancer agent toremifene (Gerits et al., 2017) and antihelminthic drug rafoxanide (Miró-Canturri et al., 2020), that target cell membrane have antibacterial activity. Previous studies demonstrated that SYTOX Green and NPN uptake assays are closely related to the permeability of bacterial membrane; Disc3(5) was used to evaluate the membrane potential depolarization, and ATP leakage was associated with membrane disturbance (Yasir et al., 2019; Elliott et al., 2020; Liu et al., 2020). Similarly, in the present study, the destruction of *S. aureus* cell membranes by PF was confirmed by increased SYTOX Green and NPN uptake and ATP efflux from the cell, as well as the loss of membrane potential determined by Disc (3)5 fluorescence. In addition, PF induced ROS accumulation, which accordingly aggravated cell membrane damage and further paralyzed bacterial homeostasis. This view was consistent with the previous observation that endogenous ROS was important for antibacterial agents (Brynildsen et al., 2013). These data indicated that bacterial cell membrane was a potential antibacterial target of PF.

In our study, PF exhibits strong antimicrobial effects against *S. aureus via* membrane disruption, but lacks significant antimicrobial activity against Gram-negative bacteria, which may be due to inner cytoplasmic membrane of Gram-negative bacteria, is surrounded by an outer membrane composed of negatively charged lipopolysaccharide (Wilson and Ruiz, 2021). Hence, it may be difficult for PF to disrupt the outer membrane, thereby transmitting to the inner cell membrane to exert antimicrobial activity. Antimicrobial agents targeting the cell membrane have attractive properties, including anti-persister potency, rapid killing, synergism with other antibiotics, and a low prospect for resistance selection (Kim et al., 2019). Our experimental results were in accordance with these properties. Transcriptome analysis showed a total of 80 upregulated DEGs, among them, *vraX* upregulation was reported associated with cell membrane or cell wall metabolism damage (Cuaron et al., 2013), and *ydfJ* belongs to a class of membrane transporter responsible for the regulation of membrane transport process (Domka et al., 2007). Besides, GO analysis also showed the upregulated genes were mainly associated with membrane and integral and intrinsic components of the membrane, further indicating that PF targets the cell membrane, resulting in compensatory upregulation of membrane-related genes. Downregulated genes, such as *fnbA*, encode fibronectin binding proteins FnBPA, which promote adherence to host tissues and biofilm development (Smith et al., 2010). The downregulation of *fnbA* indicates that PF may reduce the expression of important virulence factors and biofilm formation in *S. aureus*. Which provide further evidence that PF could inhibit biofilm formation and could be a useful therapy option for the treatment of biofilm-mediated infections.

Penfluridol is an orally bioavailable antipsychotic drug given at a dose of 20–250 mg/week. Ranjan et al. (2016) demonstrated that after intragastric administration of 10 mg/kg PF for 55 days, mice did not exhibit any significant signs of behavioral side effects or toxicity, Suggesting that PF is relatively safe even used for long-term. Similarly, in our study, PF demonstrated almost no hemolysis effect on human RBCs with limited cytotoxicity. Furthermore, from the observed synergistic antibacterial effect between PF, TC, and PMBN, it is evident that the drug combination was also an effective approach to decrease the necessary dosage of PF, thereby further reducing its cytotoxicity. Some studies have shown that PF has extensive first-pass metabolism, and once the drug reaches the systemic circulation, it is eliminated very slowly (Balant-Gorgia and Balant, 1987). This hypothesis was confirmed by our results that after intravenous, intraperitoneal, and subcutaneous administration of 10 mg/kg PF in mice, its half-life in plasma was 15.74, 14.58, and 36.86 h, respectively.

Acute bacterial skin and skin-structure infections (ABSSSI), including skin abscess, cellulitis, and wound infection, are a common reason for seeking care at emergency healthcare facilities. *Staphylococcus aureus* is the most common organism associated with these infections (Golan, 2019). To our knowledge, there has been no research on the efficacy of PF in the abscess and wound infection model with high bacterial load. In our subcutaneous abscess model, PF showed an exceedingly great effect on eliminating abscess area and bacterial load. Furthermore, PF had great antimicrobial and anti-inflammatory effects against MRSA-induced wound infection models. However, there were some limitations in this study. Despite the use of an ointment as a carrier to treat infected wounds having advantages, this preparation had some defects that may compromise compliance and effectivity of treatment. First, ointments are often oleaginous and difficult to remove, which to a certain extent affects patient compliance. Second, they may prevent excessive exudation from the wound area, which could lead to maceration of healthy skin (Lima et al., 2021). Third, although PF remains active after it is incorporated into the ointment, its stability in this formulation should be clarified in future studies. Finally, data on the release/infiltration of PF in ointments are lacking and need to be obtained in further research.

We used the acute peritonitis model as the systemic infection model to evaluate the efficacy of PF, as it is easy to master. In our *in vivo* acute toxicity test, PF did not cause mice death after intraperitoneal injection of 20 mg/kg PF for 7 days; therefore, we chose this safe dose as the therapeutic dose for the peritonitis infection model. The results indicated that PF was potent in reducing the bacterial load in the liver, kidney, and spleen, and showed a comparable efficacy to VAN. These observations suggested that PF has excellent efficacy in both local and systemic infection models in mice.

In summary, PF shows strong antimicrobial, antibiofilm, and anti-persister activity against *S. aureus*. Moreover, PF lacks resistance evolution and has low cytotoxicity as well as reasonable pharmacokinetic properties. The significant antibacterial effects of PF could be valuable in treating MRSA-related subcutaneous abscesses, skin wound infections, and peritonitis infections. These findings indicate that PF is a promising and available therapeutic candidate for MRSA infections.

## Data Availability Statement

The original contributions presented in the study are included in the article/Supplementary Material; further inquiries can be directed to the corresponding author.

## Ethics Statement

The animal study was reviewed and approved by the Ethics Committee of the Third Xiangya Hospital, Central South University (No: 2019sydw0233).

## Author Contributions

YLiu, PS, and YW designed and perfected the experiments scheme. YLiu conducted most of the experiments, analyzed the data, and composed the manuscript. LX, LC, YLi, SL, ZL, and ZH provided the reagents and some methods needed for this research. YW was responsible for supervising the entire study. All authors contributed to the article and approved the submitted version.

## Conflict of Interest

The authors declare that the research was conducted in the absence of any commercial or financial relationships that could be construed as a potential conflict of interest.

## Publisher’s Note

All claims expressed in this article are solely those of the authors and do not necessarily represent those of their affiliated organizations, or those of the publisher, the editors and the reviewers. Any product that may be evaluated in this article, or claim that may be made by its manufacturer, is not guaranteed or endorsed by the publisher.
