# Supplementary material for: Antimicrobial, Antibiofilm, and Anti-persister Activities of Penfluridol Against Staphylococcus aureus
Source: Front Microbiol. 2021 Aug 18;12:727692. doi: 10.3389/fmicb.2021.727692 (PMC8418195; doi:10.3389/fmicb.2021.727692)
Supplement: Supplementary file 1 [file Data_Sheet_1.docx]

Supplementary Material

# Supplementary Tables

**Supplementary Table 1**. Combination antimicrobial activities of penfluridol (PF) and selected drugs against *S. aureus*.

| Antibacterial agent | MIC_A_(μg/mL) | |  | MIC_B_(μg/mL) | | FICI | Outcome |
| --- | --- | --- | --- | --- | --- | --- | --- |
|  | Alone | Combination |  | Alone | Combination |  |  |
| Tetracycline | 2 | 0.5 |  | 8 | 2 | 0.5 | Synergy |
| PMBN | >512 | 64 |  | 8 | 2 | <0.375 | Synergy |
| Ceftriaxone | 64 | 32 |  | 8 | 2 | 0.75 | Addition |
| Fosfomycin | 1 | 0.5 |  | 8 | 1 | 0.625 | Addition |
| Levofloxacin | 0.125 | 0.0078 |  | 8 | 4 | 0.562 | Addition |
| Gentamicin | 64 | 64 |  | 8 | 2 | 1.25 | irrelevant |

**Supplementary Table 2.** Biofilm-forming capacity of *S. aureus.*

| Strains | A570nm (±SD) | Biofilm production |
| --- | --- | --- |
| SA 1901 | 3.282 (± 0.103) | Strong |
| SA 1902 | 2.407 (± 0.231) | Strong |
| SA 1903 | 1.487 (± 0.213) | Strong |
| SA 1904 | 3.237 (± 0.147) | Strong |
| SA 1905 | 2.667 (± 0.194) | Strong |
| SA 1906 | 2.166 (± 0.275) | Strong |
| SA 1907 | 0.971 (± 0.161) | Moderate |
| SA 1908 | 2.611 (± 0.095) | Strong |
| SA 1909 | 2.077 (± 0.155) | Strong |
| SA 1910 | 2.211 (± 0.148) | Strong |
| SA 1911 | 2.301 (± 0.185) | Strong |
| SA 1912 | 2.778 (± 0.287) | Strong |
| SA 1913 | 0.871 (± 0.136) | Moderate |
| SA 1914 | 1.962 (± 0.195) | Strong |
| ATCC 43300 | 2.709 (± 0.371) | Strong |
| ATCC 29213 | 0.907 (± 0.118) | Moderate |
| LZB1 | 3.277 (± 0.055) | Strong |
| Newman | 0.781 (± 0.145) | Moderate |
| MW2 | 1.685 (± 0.145) | Strong |
| RJ-2 | 2.887 (± 0.202) | Strong |
| SAJ1 | 2.162 (± 0.169) | Strong |
| USA300 | 2.188 (± 0.148) | Strong |

Ac=0.252

# Supplementary Figures


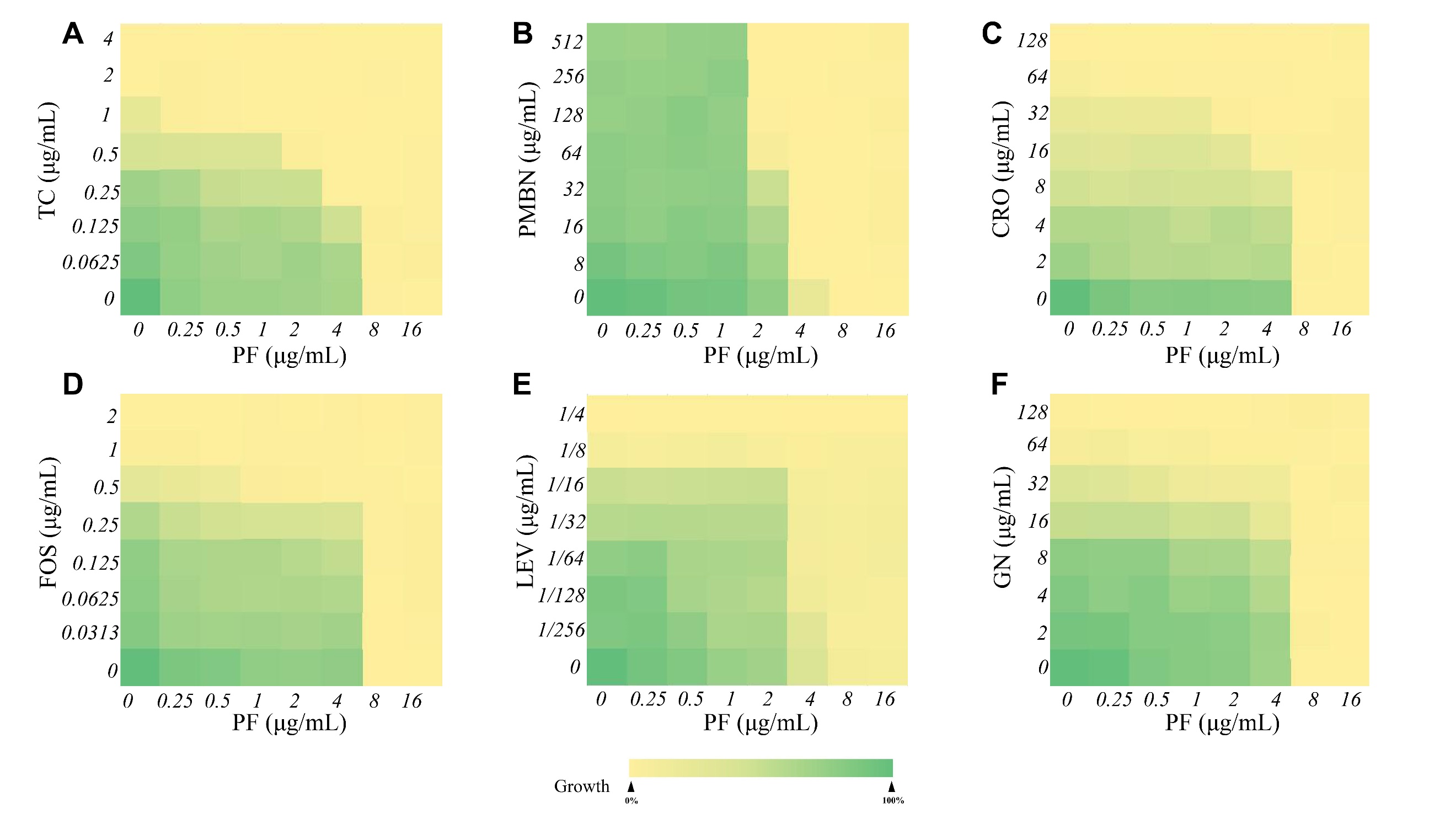


**Supplementary Figure 1.** Antibacterial effect of penfluridol (PF) combined with (A) tetracycline (TC), (B) PMBN, (C) ceftriaxone (CRO), (D) fosfomycin (FOS), (E) levofloxacin (LEV), and (F) gentamicin (GN) was determined by the checkerboard assay.

**
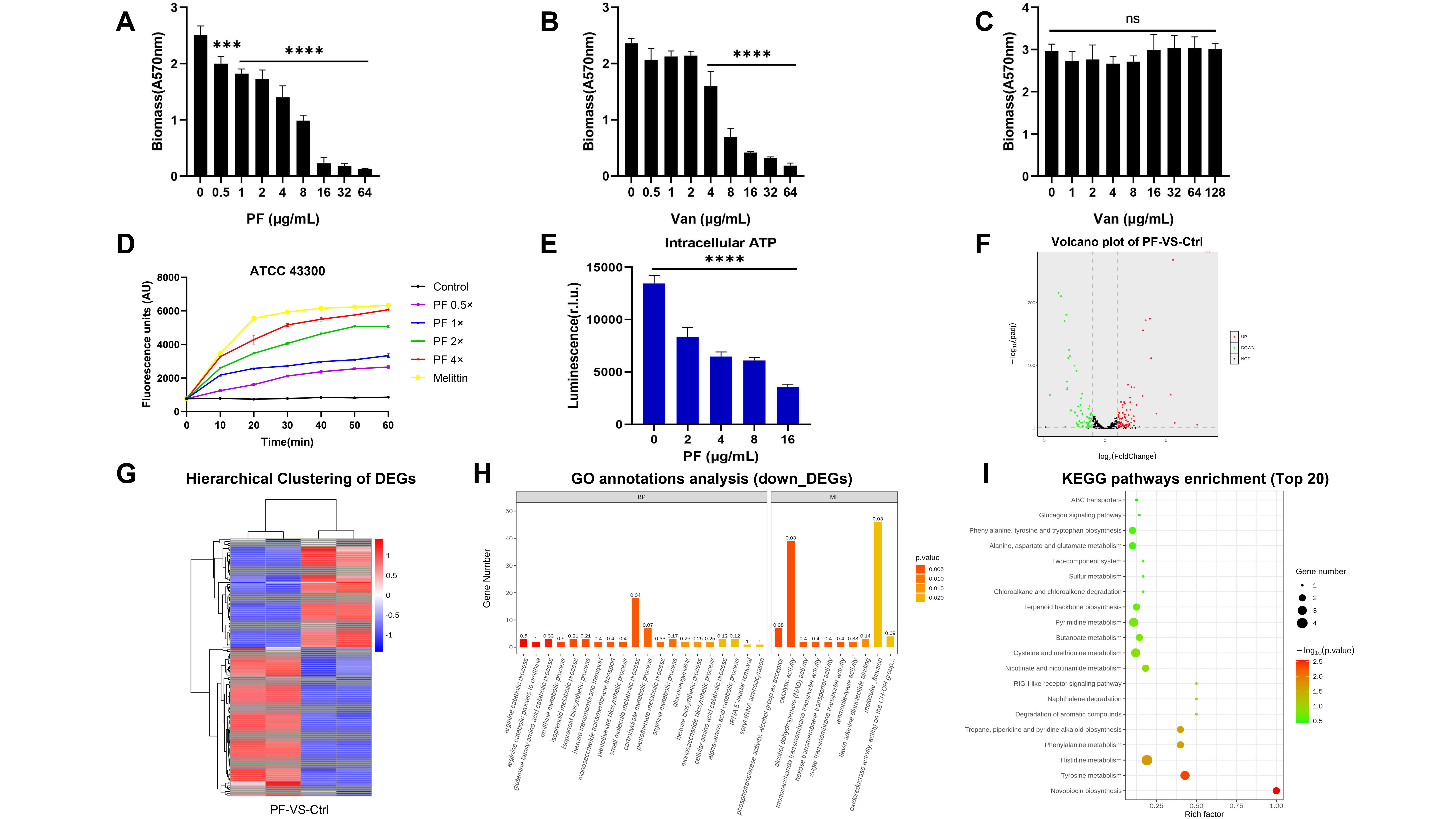
**

**Supplementary Figure 2.** Biofilm formation inhibition and RNA sequencing analysis. (A) penfluridol (PF) and (B) vancomycin (VAN) prevent biofilm formation of *S. aureus* ATCC 43300. (C) VAN cannot eradicate the 24 h biofilm of *S. aureus* ATCC 43300. (D) Uptake of SYTOX Green by *S. aureus* ATCC 43300 persister cells treated with the different PF concentrations. (E) Intracellular ATP levels decrease after PF treatment. (F) Volcano plot of upregulated and downregulated differentially expressed genes (DEGs). (G) Heatmap shows gene expression patterns. (H) GO annotation of downregulated DEGs in *S. aureus* ATCC 43300, and (I) KEGG pathway enrichment analysis of DEGs.


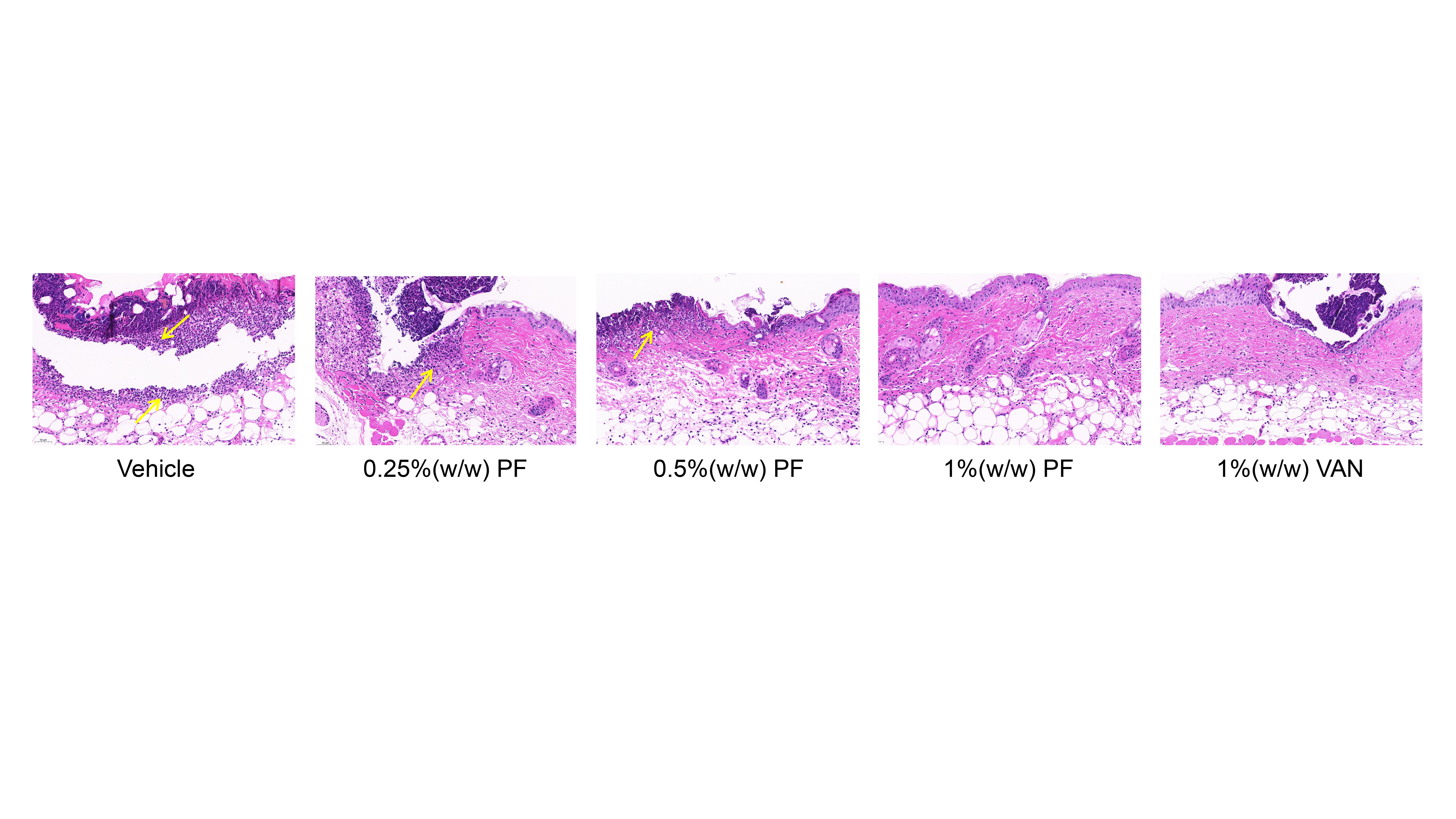


**Supplementary Figure 3.** After treatment with penfluridol (PF) or vancomycin (VAN) in wound infection model, the histopathological analysis of infected mice was evaluated by H&E staining (20×). The yellow arrow indicates extensive granulocyte infiltration.
